# Is temperature the main cause of dengue rise in non-endemic countries? The case of Argentina

**DOI:** 10.1186/1476-072X-11-26

**Published:** 2012-07-06

**Authors:** Aníbal E Carbajo, María V Cardo, Darío Vezzani

**Affiliations:** 1 Unidad de Ecología de Reservorios y Vectores de Parásitos, DEGE, FCEyN, Universidad de Buenos Aires, Ciudad Universitaria, Pabellón 2, 4° piso (C1428EHA), Buenos Aires, Argentina; 2 Ecología de Enfermedades Transmitidas por Vectores (EETV), Instituto de Investigaciones e Ingeniería Ambiental (3iA) Universidad Nacional de General San Martín, Peatonal Belgrano 3563 (1650), San Martín, Prov. de Buenos Aires, Argentina

## Abstract

**Background:**

Dengue cases have increased during the last decades, particularly in non-endemic areas, and Argentina was no exception in the southern transmission fringe. Although temperature rise has been blamed for this, human population growth, increased travel and inefficient vector control may also be implicated. The relative contribution of geographic, demographic and climatic of variables on the occurrence of dengue cases was evaluated.

**Methods:**

According to dengue history in the country, the study was divided in two decades, a first decade corresponding to the reemergence of the disease and the second including several epidemics. Annual dengue risk was modeled by a temperature-based mechanistic model as annual days of possible transmission. The spatial distribution of dengue occurrence was modeled as a function of the output of the mechanistic model, climatic, geographic and demographic variables for both decades.

**Results:**

According to the temperature-based model dengue risk increased between the two decades, and epidemics of the last decade coincided with high annual risk. Dengue spatial occurrence was best modeled by a combination of climatic, demographic and geographic variables and province as a grouping factor. It was positively associated with days of possible transmission, human population number, population fall and distance to water bodies. When considered separately, the classification performance of demographic variables was higher than that of climatic and geographic variables.

**Conclusions:**

Temperature, though useful to estimate annual transmission risk, does not fully describe the distribution of dengue occurrence at the country scale. Indeed, when taken separately, climatic variables performed worse than geographic or demographic variables. A combination of the three types was best for this task.

## Background

The incidence of dengue has grown dramatically around the world in recent decades, with 2.5 billion people–two-fifths of the world’s population–currently at risk. The World Health Organization estimates there may be 50 million dengue infections worldwide every year [1]. The number of cases and countries affected is increasing; more frequent and larger epidemics associated with more severe disease are occurring [2]. The hypothetical causes of dengue increase include a combination of multiple factors, namely the range expansion of its primary vector (the mosquito *Aedes aegypti*), inefficient vector control, human population growth, unplanned urbanization, increasing movement of people incubating the virus by plane, genetic changes in circulating or introduced viruses and modulating climatic factors [2-4].

The study of dengue distribution has been traditionally addressed by two broad approaches: theoretical models (also known as mathematical, mechanistic or biological models) and empirical models (also known as statistical models) [5]. The former make use of mosquito life tables and vectorial capacity including several bionomic parameters (e.g. [6,7]). They consider variables which are physiologically related to transmission (e.g. rates of development, food availability, life expectancy), but are difficult to implement at the regional scale without simplification, because parameters must be estimated for different locations and times, otherwise assumed constant. On the other hand, empirical approaches relate the distribution of cases to climatic and other environmental variables (e.g. [8-10]). They model the outcome of several factors that drive the occurrence of the disease, although may sometimes associate the distribution of cases with spurious variables [11]. Each approach has its own advantages and drawbacks for predictive purposes. Theoretical models may disregard variables that empirical approaches find essential, leading to an overestimation of dengue expansion. Empirical models rely on the current distribution of cases, which may not necessarily extend to the entire potential or historic area, and thus might underestimate the future distribution of the disease [5].

At the global scale, dengue transmission studies based on climate have pointed out a potential rise or expansion of transmission under several global warming scenarios [6-8,12]. These findings have shown that a small increase of 1°C in temperature can lead to substantial increases in transmission potential [13]. However, the influence of the past century’s temperature rise (≈0.5°C) on the widespread reemergence of this and other vector-borne diseases remains undetermined [7]. Despite overall increases in temperature over the past century, a contraction of the geographic distribution of dengue has occurred; e.g. in the southern states of North America, much of Australia, parts of southern Europe, Japan, China and South Africa. On the other hand, several countries have recently reported dengue transmission for the first time, but it is unclear whether this represents a true geographic spread or increased awareness and reporting [13]. Some authors postulate that climate has rarely been the principal determinant of the distribution range of diseases [4,14]. In areas located at the fringe of transmission in which outbreaks occur typically during the warm season, temperature is undoubtedly of major importance. However, in hyperendemic tropical and subtropical regions, disease transmission may be saturated, and patterns of human migration of susceptible individuals are likely to be more relevant to overall transmission than climatic factors [7]. It has been speculated that in such areas dengue increase was the result of rapid urbanization and increased international travel [2,15]. In view of this, distribution models could be improved by considering non-climatic factors such as human population density, mosquito control, water storage systems, travel and migration, socio-economic influences, immunity patterns and drug resistance effects [13].

In Argentina, since dengue reemergence in 1998, some sporadic epidemics of less than 1,500 autochtonous cases affecting a few districts have occurred in subtropical provinces [16]. In 2009 the biggest outbreak known to date occurred (> 25,000 autochthonous cases), affecting almost half of the country and reaching temperate latitudes [17,18]. Some global models described the situation in Argentina until 2007 appropriately but failed to predict the transmission spread toward temperate latitudes [9]. Moreover, the 2009 epidemic can only be predicted by climate change projections more than 50 years ahead, or following a 2°C rise in global temperature [6-8,12]. This fact evidenced the limitations of both global models and climate change forecasting for assessing local situations. Prior to this epidemic, a local risk model calibrated with historical data had attained better results for our country than global approaches [19].

The purpose of this paper was to study the factors associated with the occurrence of dengue in Argentina. The relative importance of geographic, climatic, and demographic variables was compared to assess whether temperature was the main factor associated with the spatio-temporal distribution of cases.

## Results

**Figure 1:**
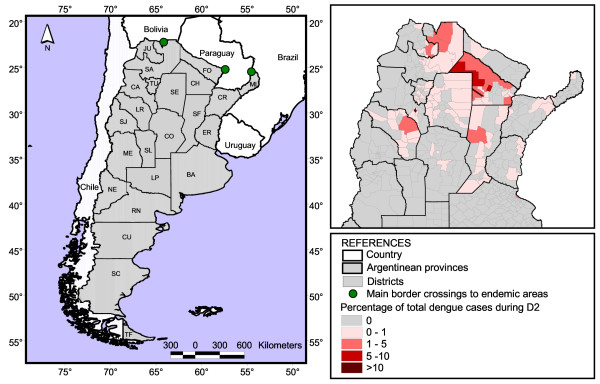
**Study area and dengue cases by district.** Left, Country provinces. JU, Jujuy; SA, Salta; FO, Formosa; SE, Santiago del Estero; TU, Tucumán; LR, La Rioja; SJ, San Juan; CA, Catamarca; SL, San Luis; CO, Córdoba; SF, Santa Fe; CH, Chaco; CR, Corrientes; ER, Entre Ríos; ME, Mendoza; NE, Neuquén; RN, Río Negro; LP, La Pampa; BA, Buenos Aires; Cu, Chubut; SC, Santa Cruz; TF, Tierra del Fuego. Right, Percentage of reported autochthonous dengue cases per district throughout Argentina in the period July 2001–June 2011 (D2).

At the country level, the percentage of districts recording autochthonous dengue transmission increased from 1.2% (6/503) in D1 to 19.5% (98/503) in D2. Such districts were located mainly in the subtropical region and extended toward temperate latitudes down to Buenos Aires Province (Figure 1). During D2, 8 districts presented more than 1000 confirmed cases each (5 in Chaco, 2 in Salta and 1 in Catamarca), representing 69% (20,376/29,510) of the total confirmed cases in the country. The highest number of cases was recorded in one district of Catamarca Province (8,861 cases) followed by other of Chaco Province (3,148).

### Mechanistic model

Mean temperature per district was 0.24°C higher in D2 than in D1 (paired test t = 54.29; df 479, p < 0.0001). Comparing both decades, dengue risk increased in 12.5 annual DPT (paired test t = 46.36; df 479, p < 0.0001).

**Figure 2:**
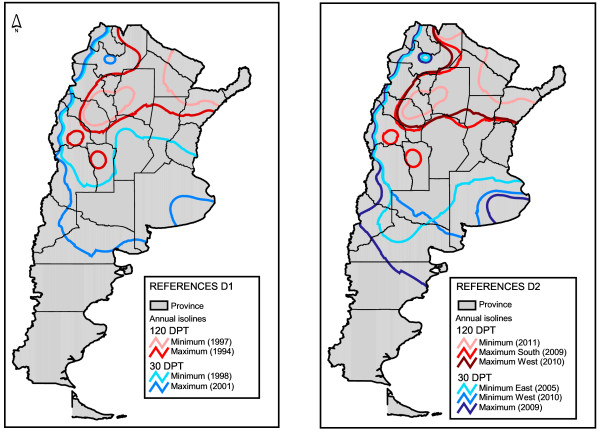
**Dengue transmission risk according to DPT.** Dengue transmission risk maps as isolines for the annual days of possible transmission (DPT). Left, isolines for the period 1991–2001 (D1). Right, isolines for the period 2001–2011 (D2). Only the isolines of extreme years are shown. When the maximum or minimum differs by zone, both isolines are shown. Each year spans from July of the previous year to June of the indicated year (e.g. 2000 includes 365 days from 1 July 1999 to 30 June 2000).

The DPT showed a dynamic pattern throughout the study period. According to the maximum latitude reached by annual isolines, the maximum transmission risk during D1 was observed in 2001 and 1994 for temperate and subtropical regions, respectively (Figure 2). Maximum risk in D2 was registered during 2009 in temperate zones whereas in subtropical zones it was during 2009 and 2010 (maximum reach to the south and west, respectively). Both in temperate and subtropical areas the overall maximum risk occurred in D2 (2009 and 2010), whereas the minimum was observed in D1 (1997 and 1998) (Figure 2).

**Figure 3:**
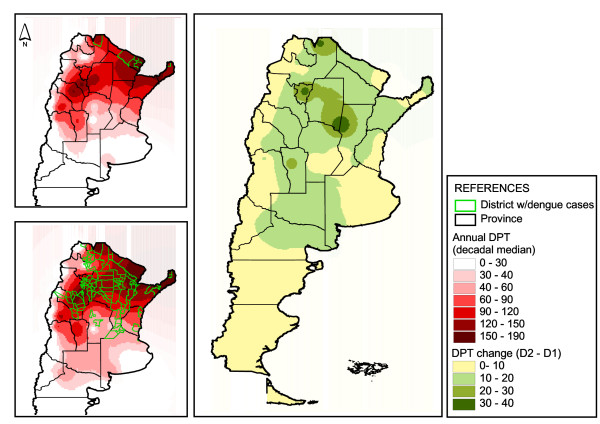
**Dengue transmission risk change according to DPT.** Right, Increase of dengue risk from D1 to D2 based on days of possible transmission (DPT_D2_-DPT_D1_). Upper left, DPT for D1. Lower left, DPT for D2.

The DPT map that resulted from subtracting DPT_D2_-DPT_D1_ showed an increase in risk throughout Argentina (Figure 3). In particular, hot areas in the north and center of the country were observed. For example, dengue risk between decades increased more in La Pampa than in southern Formosa, despite the fact that the effective risk was higher in the latter province. Epidemic transmission during D1 was apparently limited by 120 DPT (Figure 3). In D2, autochthonous cases occurred up to 50 DPT, and epidemic episodes in Catamarca and Chaco provinces developed with more than 90 DPT (Figure 3). Some districts located in the west of Jujuy and La Rioja provinces were excluded from this interpretation because their DPT range includes extremely low values from the Andean region and the cases occurred in the east.

### Statistical model

Major steps in model selection and partial best models for each type of variables are shown in Table 1. The random term ‘province’ was retained in the model, confirming a significant correlation among districts within each province. As shown by the selected final model (Table 2), the probability of occurrence of a dengue case in a given district was positively associated with distance to water courses (DI.wa), human population (log scale, lo.po), and DPT, and negatively related with the percentage of human population change (prc). Classification effectiveness of the model was 71% better than random (K = 0.71; range of agreement: ‘substantial’). Specificity of the model was 0.92, sensitivity was 0.83, and overall correct classification was 0.90.

**Table 1:** Statistical model variables selection

**Model**	**Fixed variables**	**Kappa**	**AIC**
Null w/o random	1	0	487.9
Null	1	0.54	373.5
Full	DI.wa + prc + lo.po + MinT + WI + DPT	0.69	293.9
Final	DI.wa + prc + lo.po + DPT	0.71	292.0
Final–DPT	DI.wa + prc + lo.po	0.66	302.9
Partial geographic	DI.wa + DI.en + AL	0.62	328.6
Partial climatic	MeanT + WI	0.61	342.9
Partial demographic	prc + lo.po	0.68	308.9

Model selection for the probability of occurrence of a dengue case in Argentina between 2001 and 2011. All models except indicated contain the grouping variable Province as random factor. See Table 4 for variables abbreviations.

**Table 2:** Final statistical model parameters

**Variable**	**B**	**SE**	**t value**
intercept	−3.2143	0.6465	−4.972 ***
DI.wa^2^	0.0035	0.0013	2.737 **
Prc	−3.8063	1.4448	−2.635 **
Lo.po	1.2311	0.1847	6.666 ***
DPT	0.0265	0.0077	3.419 ***

Generalized Linear Mixed Model parameter (B), standard error (SE), and t value for each variable included in the selected model for the occurrence of dengue in Argentina between 2001 and 2011. See Table 4 for variables abbreviations.

*** Significant at P < 0.001; ** P < 0.01.

**Figure 4:**
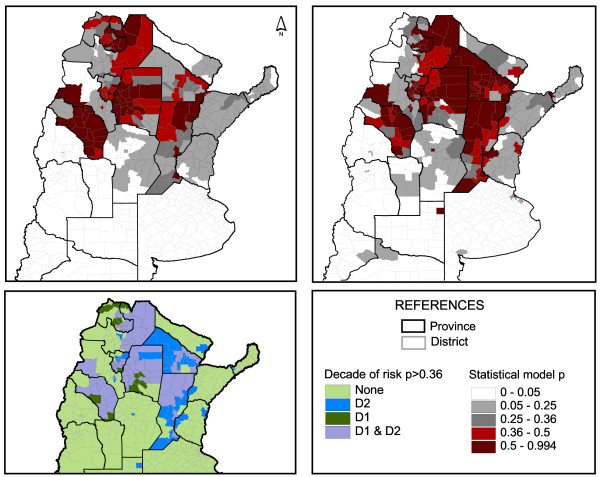
**Dengue transmission risk according to the statistical model.** Model prediction of dengue risk for D1 (upper left) and D2 (upper right). Districts in red (>0.36) and dark red (>0.5) are classified as positive for dengue by the model. Lower left, districts under risk by decade.

The cut-off point (cp) was 0.36; i.e. a district with an occurrence probability ≥0.36 should indicate the presence of at least one dengue case, whereas lower values suggest that dengue occurrence is unlikely. The number of districts classified as negative in both decades was 355, whereas the same applied to positives was 70. More districts were classified as positive in D2 than in D1; 112 and 83, respectively (Figure 4). Of those, 13 districts which were classified as positive in D1 were considered negative by the model in D2, and only one of such districts presented cases in D2. Of 42 districts becoming at risk from D1 to D2, 29 (69%) presented cases in D2. Districts presenting increased dengue risk in D2 were mainly located in Chaco and Santa Fe provinces, but also spread to higher latitudes in La Pampa Province and the Federal District in Buenos Aires Province (Figure 4). In D2, 83% (81/98) of the districts with confirmed cases were correctly classified as positive. Remarkable exceptions were some districts in Misiones and Formosa provinces, which are closest to main neighboring endemic areas. In D1, however, only 1 out of 6 positive districts were correctly classified by the model. Notwithstanding, validation power is poor due to the low number of districts with cases during D1. The total population at risk in D1 was 6,517,615 and increased to 14,052,262 in D2. This responds mainly to the addition of big cities, like the Federal District and Córdoba City, adding 4,660,000 inhabitants.

**Figure 5:**
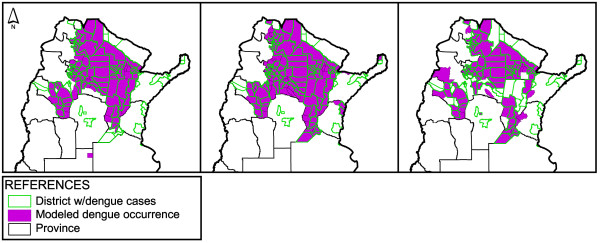
**Dengue occurrence according to the partial models.** Predicted dengue occurrence according to each of the partial models. Left, geographic; Middle, climatic; Right, demographic.

The exclusion of the variable DPT from the selected final model resulted in a decrease of the K value, confirming its association with dengue occurrence (Table 1). The probability of dengue occurrence according to each subset of explanatory variables model is shown in Figure 5. Partial best models (geographic, climatic, and demographic) yielded K values within the ‘substantial’ range of agreement (Table 1). Among the three, the climatic model presented the highest AIC with the lowest classification power.

## Discussion

The mean annual temperature in Argentina has increased during the last decade. Accordingly, the temperature-based mechanistic model showed an increased dengue risk, especially in Tucumán, northern Salta and Córdoba provinces. At the yearly scale, the maximum risk according to DPT coincided with the greatest epidemic in the country. Furthermore, the years 2004 and 2010 (which followed 2009 in number of cases) presented more extreme 120 DPT isolines to the west than 2009. However, the association between DPT and dengue cases was not observed during D1 as the reemergence of dengue in 1998 did not present increased DPT. The temperature/EIP relationship has been mentioned to account for approximately 75–85% of the variability in transmission elsewhere [7].

The selected statistical model included environmental and demographic variables. It best described the spatial probability of occurrence of dengue cases in association with temperature (expressed as days of possible transmission), human population number and change and distance to water courses. The latter variable may reflect district dryness, implying different water storage behavior associated with variable breeding conditions for the vector [14,20,21]. Although precipitation may be a better index of this behavior, that variable should be excluded from the modeling due to its high correlation with temperature. Higher human population may be related to increased virus pressure resulting from higher travel rates [22]. In addition, bigger cities are associated with more susceptible and overcrowded population and a greater availability of containers acting as breeding habitat for *Ae. aegypti* as a consequence of uncontrolled urbanization. Argentina is until now an epidemic country, therefore transmission relies on the arrival of viremic individuals from endemic areas. Highly populated areas are expected to experience more travel flux with endemic countries and consequently higher virus pressure, redounding in higher transmission rates. It is interesting to note that a significant association with human population change was also observed. However, the sign of this relation was opposite to the expected according to Wilder-Smith and Gubler [4], because cases were associated with districts in which population fell. The majority of cases during the 2009 epidemic occurred in provinces where population decreased between 2001 and 2011. We believe that the fall in population may be related to laborer migration to highly populated areas, where virus pressure from endemic countries is higher [22]. This in turn might raise the probability of carrying the virus back when visiting family members. On the other hand, a reduction in population may be associated with emigration from poor living conditions given that poverty has been previously related to more successful vector breeding [21].

The change in risk between decades 1 and 2 predicted by the statistical model showed districts that stopped being risky in the west and others that became risky in the center-east. The latter area was mainly affected by the 2009 epidemic. The absence of risk in districts of Formosa and Misiones provinces located close to the bordering endemic areas is surprising. A high immunization rate of the human population does not seem a plausible explanation, since epidemics in analogous districts close to the endemic border in Salta Province have frequently occurred. Asymmetries in case reporting or in the efficiency of control measures (on the vector, the transmission or even the information) at the province level should not be discarded, as provinces were detected as a good random grouping factor in explaining dengue occurrence. It is worth mentioning that municipalities are responsible for control strategies, while provinces supervise and coordinate the actions and the national service intervenes only occasionally [23]. There is a remarkable lack of coherence and coordination among the different health departments at the province level in Argentina, both for the taking and treatment of the data. Modeling precision and risk forecasting would be greatly improved if national health authorities achieve coherence in data gathering and processing throughout the country.

Although the final statistical model included DPT as a predictor of the spatial distribution of dengue cases, interestingly the partial climatic model was the worst in this task. The partial statistical models with climatic and geographic variables showed similar classification power. There is confusion among these variables inherent to Argentinean territory because the distance to endemic areas is inversely correlated to temperature. The effect of virus pressure, related to the distance to endemic areas, might therefore be confused with higher temperatures, which favor transmission. Temperature was selected in the partial model instead of DPT. Although these two variables were highly correlated, temperature was excluded from the full model due to its high variance inflation factor. This probably responded to temperature being correlated to several other non climatic variables. The partial models pointed out the importance of the initial variable pool for model development. Significant results may be obtained within a subset of variables leading to biased conclusions.

Using global models for local control of dengue has been questioned [20]. Although they may depict the general situation, they do not have the necessary detail to drive control strategies at the country scale. Models should not only include local and historical data but also consider local processes that might work differently among regions. Climate change would affect the extent of the transmission area of vector borne diseases, especially at the distribution fringe [6-8]. Both models considered herein give a more detailed view of risk behavior under changes that have taken place between two decades coinciding with demographic and temperature changes. In particular, it is interesting to note that the expansion of the transmission area has occurred mainly southwards. This expansion may be explained better by temperature in some areas and by demography in others. For example, Santa Fe Province presented plenty of districts with cases in D2. The northwestern districts were risky according to the statistical model during both decades, but presented an important rise in DPT from D1 to D2. On the other hand, the southern districts showed little DPT change, but became risky according to the statistical model in D2. Our hypothesis, confirmed by the partial model analysis, is that the higher virus pressure in more populated areas compensates for the less favorable temperature conditions in the center of the country.

In addition to the reported factors, the increased dengue occurrence could be influenced by the dengue rise at the continental or global scales and by the growth of international travel in the last decades, which may in turn augment virus importation into epidemic countries or increment virus epidemic potential [15,20,24,25]. These potential factors would enhance virus pressure at local levels. Other local factors such as the availability of breeding sites and shelter for the vector, poverty, entomological and medical surveillance and control measures were not considered in our model and may be significant for further explaining the spatial distribution of cases. Also, virus pressure from endemic neighboring countries could not be included in the analysis because reliable data on migration among countries is not available.

## Conclusions

During the last decade, dengue epidemics in Argentina were associated with high risk modeled as a function of temperature (annual days of possible transmission–DPT).

The distribution of dengue occurrence was associated with higher DPT, higher human population, population decrease and higher distance to water bodies, with ‘province’ as a significant grouping factor. Climate cannot be considered the main predictor of dengue distribution, as in fact demographic variables performed better. The southward expansion of the transmission fringe may have been driven by temperature or demography depending on the area.

## Methods

Argentina extends from latitudes 22 to 55 S, presenting subtropical and temperate regions. All neighboring countries to the north (Brazil, Paraguay and Bolivia) are dengue endemic areas, and Argentina represents the southern limit of dengue transmission in South America, with only epidemic outbreaks until the present, concentrated in the warm season. This epidemic nature and the low number of annual cases preclude the development of a temporal dynamical model (e.g. [26-29]).

Information on dengue cases was available with varying detail from locality to district and day to year [17,30]. All cases were compiled to the broader scale: district and year (Table 3).

**Table 3:** Dengue annual cases in Argentina

**Year**	**1998**	**1999**	**2000**	**2001**	**2002**	**2003**	**2004**	**2005**	**2006**	**2007**	**2008**	**2009**	**2010**	**2011**
Cases	330	0	445	0	204	91	1493	0	175	173	40	25897	1185	252
Number of affected districts	1	0	5	0	1	4	5	0	3	5	1	84	3	2

Number of dengue autochthonous clinical cases and positive districts throughout the country per year. No cases were reported between 1991 and 1997. Data source 1998–2009 [17], 2010–2011 [30].

Ancillary demographic variables were available at the decadal time scale [31,32]. Therefore, data was also compiled in two decades that represented distinct periods: dengue reemergence (July 1991–June 2001) herein D1, and dengue epidemics (July 2001–June 2011) herein D2.

### Mechanistic model

Annual risk of dengue transmission was modeled based on temperature. The number of days of possible transmission per year (DPT) was calculated as follows The extrinsic incubation period (EIP) of the dengue virus in the mosquito *Ae. aegypti* is the lapse from ingestion of infected blood to the virus transmission in a subsequent feed. This period varies as a function of temperature [33,34]. If ambient temperature is low, mosquitoes are unlikely to survive long enough to become infectious and transmit the disease. Therefore, the duration of the EIP and the survival of vectors are key factors in determining dengue transmission risk [35]. To estimate such risk, the number of days per year that the EIP could be completed before the vector died was calculated using a mathematical model based on enzyme kinetics that relates the virus development to air temperature [35-37]. Based on a previous study, the survival of *Ae. aegypti* in Argentina was fixed to 15 days [19]. The model developed by Jetten and Focks [6] was modified to estimate the completion of EIP in each hour:

(1) $$r\left(T_{h}\right)=\left(p_{\left(25{}^{\circ}C\right)}\left(T_{h}/298\right)e^{x1}\right)/\left(1+e^{x2}\right)$$

where

(2) $$\begin{array}{l} x_{1}=\Delta H{*}_{A}/R\left(\left(1/298\right)-\left(1/T_{h}\right)\right)\;\mathrm{and}\\ x_{2}=\Delta H{*}_{H}/R\left(\left(1/T_{0.5H}\right)-\left(1/T_{h}\right)\right) \end{array}$$

r(T_h_) represents the development rate (hr^−1^) at temperature T (°K) at hour h, p_(25°C)_ is the development rate (hr^−1^) at 25°C assuming no temperature inactivation of the critical enzyme, ΔH*_A_ is the enthalpy of activation of the reaction that is catalyzed by the enzyme (cal/mol), ΔH*_H_ is the enthalpy change associated with high temperature inactivation of the enzyme (cal/mol), T_0.5H_ is the temperature (°K) where 50% of the enzyme is inactivated by high temperature, R is the universal gas constant (1.987 cal/mol/°C), and CD, represents cumulative development. The parameters were modified to match the EIP given in Focks et al. [38], and to include a temperature of 40°C as a limit for mosquito survival (p_(25°)_ = 0.003; ΔH*_A_ = 13000; ΔH*_H_ = 110000; T_0.5H_ = 313). Considering that daily temperature behavior affects virus development [39], the daily EIP was calculated on a two-hour basis through the asymmetric interpolation of minimum and maximum daily temperatures. A linear rise between 6 am and 2 pm (i.e. the time of minimum and maximum temperature, respectively) and a linear fall from 2 pm to 6 am of the following day was used. Climatic data was obtained from 38 meteorological stations throughout Argentina [40]. It was assumed that the vector was potentially present in all localities, that it had acquired the virus by biting a viremic person and that its life expectancy was constant through the years and all over the country. The EIP completion at each locality was calculated, by adding the daily EIP completed in each day of the year plus the 14 subsequent days. If the result was >0.99, a value of 1 was given to the starting day, otherwise a 0 was assigned. The annual sum of days in which the EIP could be completed was defined as DPT. The DPT for each meteorological station and year were interpolated for the whole country by the inverse weighted distance method on a 15 km square cell grid [41]. Considering that epidemics occur during the warm season, dengue cases for a given year were paired with the DPT of the period from the previous July to June of the corresponding year. Each period was named after the ending year (e.g. 7/1/1997–6/30/1998 was referred to as 1998). Maps were developed for each one-year period between July 1991 and June 2011. Maps were built for both decades using the median DPT of the 10 years. Changes in temperature and DPT per district between both decades were evaluated with paired t-tests, and transmission thresholds in DPT were visually estimated. Annual risk was represented by isolines of 30 and 120 DPT. The change in risk between both decades was compared by subtracting both maps. All analyses were performed in ArcView 3.2 [42].

### Statistical model

Generalized Linear Mixed Models (GLMM) were used to model the probability of dengue occurrence based on the cases registered during D2 which included the 2009 epidemic plus 8 smaller outbreaks. Modeling the number or the incidence of dengue cases was too ambitious given the poor quality of the available data. Whereas the records of autochthonous cases were reliable, the number of cases per district and the absence of cases were not. There were many districts without cases that were suspected positive. There were also 29 districts with one autochthonous case, in which transmission was postulated despite the number of cases was probably underestimated. Therefore, a binomial GLMM was chosen to model dengue occurrence. These models allow the treatment of data with errors that do not follow a normal distribution, and the inclusion of random terms (grouping variables) to account for temporal or spatial correlation in the data [43].

A large area beyond the distribution limits of *Ae. aegypti,* comprising the 24 districts of the southernmost provinces, was excluded to better model the northern half of the country. The probability of dengue occurrence in each district was modeled with presence/absence of cases as the response variable, binomial error distribution, and logistic function as a link between the response variable and the lineal predictor [44]. A preliminary pool of variables as diverse as possible was selected. Afterwards, the selection was restricted by regional availability, grain resolution (lower than 15 km pixel) and temporal resolution (year to decade). Variables were then analyzed for collinearity. From each pair of highly correlated variables (>0.9) the more widely used variable was kept (e.g. temperature prior to number of frost days). Twelve explanatory variables were included in the model, divided in geographic (AR, AL, ALsd, DI.wa, DI.en), climatic (MeanT, MinT, PP, DE, SL, WI), and demographic (lo.po, prc) variables (Table 4) [31,32,40,45,46]. The DPT resulting from the mechanistic model was included as an explanatory layer in the current statistical model. All variables were calculated for both decades. Model selection and parameters estimation was based on values of D2 (see below). Daily values of climatic variables at each meteorological station were downloaded from NOAA Satellite and Information Service [40]. Mean values for each decade were calculated and interpolated using the kriging method [41] on a 15 km square cell grid; mean values for each district were then extracted for DPT, climatic, and geographic variables. Demographic variables were calculated from three national censuses performed in 1991, 2001, and 2010 [31,32]. The log transformed population in 2001 and 2010 were used to characterize D1 and D2, respectively. Population density was not included because it was very skewed. The proportion of human population change between decades was calculated as (pop. D2–pop. D1)/pop. D2. Other demographic variables gathered in the mentioned censuses (percentage of homes without sewage service, regular garbage collection and water network) were not available at the spatial scale required. All variables (x_i_) were centered and squared, and entered in the model as x_i_, x_i_^2^, and x_i_ + x_i_^2^.

**Table 4:** Statistical model explanatory variables

**Variable group**	**Variable**	**Description**	**Source**	**Units**	**Cell approx. side (km)**
Geographic	AR	Area of each district	[46]	m^2^	-
	AL	Mean district elevation above sea level	[45]	m	1
	ALsd	Standard deviation of altitude of all pixels within a district	[45]	m	1
	DI.wa	Distance to the nearest water body or course (excluding the sea)	[46]	km	13
	DI.en	Distance to nearest border crossing to endemic area	[46]	km	13
Climatic	MeanT	Mean annual temperature	[40]	°C	15
	MinT	Minimum annual temperature	[40]	°C	15
	PP	Mean annual cumulative precipitation	[40]	mm	15
	DE	Mean annual dew point	[40]	°C	15
	SL	Mean annual sea level pressure	[40]	hp	15
	WI	Mean annual windspeed	[40]	m/s	15
	DPT	Days of possible transmission	[40]	days	15
Demographic	prc	Percentage of population change per district	[31,32]	---	---
	lo.po	Logarithm of population per district	[31,32]	Log (people)	---

Explanatory variables included in Generalized Linear Mixed Models for the occurrence of dengue in Argentina between 2001 and 2011.

To identify the best model, the largest set of explanatory variables without collinearity was first identified. Collinearity among variables was assessed with the variance inflation factors (VIFs) [47]. If any of the VIF values was higher than 5, indicating multicollinearity [48], the variable with the highest VIF was dropped, the VIFs recalculated and the process repeated until all values were lower than 5. Once the largest set of explanatory variables was identified, ‘province’ was added as a random intercept to assess whether it improved the model. The resulting model at this step was considered the full model and a backward stepwise manual procedure was performed to evaluate which variables to retain. Decision rules for random factor addition and variables dropping were based on the Akaike’s information criterion (AIC) [49]: the model that yielded the lowest AIC was selected from all possible models [50].

To evaluate and compare the contribution of each group of variables (i.e. geographic, climatic, and demographic) to the occurrence of dengue, the same protocol used for obtaining the full model was applied to each subset of variables separately and three partial best models were obtained.

As the output variable of the binomial model lies between 0 and 1 a threshold probability must be selected to distinguish positive from negative (dengue presence and absence, respectively). All possible cut-off points from 0.01 to 0.99 were assessed to select an optimum cut-off point (cp) which maximized the classification effectiveness of the model. This was evaluated by applying the Kappa index (K) to assess improvement of classification of the model over chance, with the following ranges of agreement: poor K<0; slight 0≤K≤0.2; fair 0.2<K≤0.4; moderate 0.4<K≤0.6; substantial 0.6<K≤0.8; and almost perfect 0.8<K≤1 [51,52]. The Kappa index overcomes the problem of unequal number of presences and absences [53]. For the final selected model, we also calculated (a) specificity: the proportion of negatives correctly identified, (b) sensitivity: the equivalent for positives, and (c) correct classification: the proportion of well classified negatives and positives.

Finally, once the best model using explanatory variables from D2 was obtained, the same model and parameters were run with climatic and demographic variables corresponding to D1. The correct classification of the districts with cases during D1 was evaluated. The final decadal risk maps for both decades were built applying the GLMM formula to each district in the GIS. The softwares R 2.13.0 with lme4 and Design packages, and Arcview 3.2 were used for modeling and mapping, respectively [42,54].

## Abbreviations

cp: Cut-off point. Probability used as a cut off to assign presence or absence of dengue cases, based on classification accuracy; AIC: Akaike’s information criterion; D1: Decade 1, July 1991–June 2001; D2: Decade 2, July 2001–June 2010; DPT: Annual days of possible transmission, according to a daily temperature dependent model; EIP: Extrinsic incubation period. Time in days needed for the virus to develop in the mosquito and being able to be transmitted; GLMM: Generalized linear mixed models; K: Kappa index; VIF: Variance inflation factor.

## Competing interests

The authors declare that they have no competing interests.

## Authors’ contributions

AEC designed the study and made the maps. MVC gathered data. All authors contributed to the analysis of the study and writing of the paper. They read and approved the final manuscript.
